# A224 USTEKINUMAB EFFECTIVENESS AND SAFETY IN PEDIATRIC PERIANAL CROHN’S DISEASE, A CASE REPORT AND LITERATURE REVIEW

**DOI:** 10.1093/jcag/gwac036.224

**Published:** 2023-03-07

**Authors:** M Abu Abthan, P Church

**Affiliations:** Pediatric GI, The Hospital for Sick Children, Toronto, Canada

## Abstract

**Background:**

Crohn’s disease (CD) is a chronic, relapsing, destructive, and inflammatory disease with penetrating, stricturing, or just inflammatory behavior that might affect the gastrointestinal tract or present with extraintestinal manifestations. Perianal fistulizing CD remains a challenging condition, with anti-tumor necrosis factor alpha (Anti-TNF) being the preferred, and effective therapy in the majority of cases.

**Purpose:**

Ustekinumab (UST) is in off-label use for refractory CD in children especially those who don’t show a response to anti-TNF. However, data on safety and efficacy in children is limited.

**Method:**

Our patient is 15-year-old girl, started to have a recurrent perianal and labial abscess at age of 11 yr. Despite having a normal upper and lower colonoscopy in the first place, pathology examination showed mild regenerative glandular alterations in the rectum. However, she continued to have recurrent peri-anal and vulval abscesses, in November 2016, April 2017, June 2017, and February 2018, which required multiple incision, drainage, fistulotomy as well as antibiotics therapy. Further evaluation revealed an isolated perianal Crohn’s disease for which she was commenced to Adalimumab, and despite optimal dosing, she continued to have an active disease with partial response evidenced by biochemical and radiological findings. The decision was made to shift from Adalimumab to weight-based UST doses based on the promising results in adult CD.

**Result(s):**

Our patient was followed frequently to ensure good therapeutic drug level, inflammatory markers improved from the maximum during her active disease till the last readings (56 weeks after starting UST), ESR (2-34 mm/hr) of 46 to 15 and CRP (1.0-1.7) of 21 to 0.2. Albumin and fecal calprotectin remain within normal range throughout her disease course. Repeated MRI showed Interval resolution of ischiorectal/ischioanal collection, almost complete resolution of transsphincteric fistulous tract while another intersphincteric fistula remained unchanged. Nonetheless, our patient reported a significant improvement in her perianal pain and weight gain of 6 kg during the interval assessment at 56 weeks of therapy.

**Conclusion(s):**

UST therapy shows a modest response in treating perianal CD in adult population, *Godoy et al* reported 59.3% of their cohort showed symptomatic improvement in fistula drainage and discomfort at 6 months follow-up. While at 12 months all patients had a symptomatic response with 22.2% achieving symptomatic remission. Moreover, a systematic review included a total of 774 patients with active perianal disease received UST, and 71 received placebo. At 12 months, 152 patients had a follow-up, out of which 85 (53.9%) achieved response, and 44 out of 105 (41.9%) achieved remission. *Kim et al* reported 13 pediatric patients treated with UST, 8 (38.5%) of which achieved PCDAI reemission.

The safety and efficacy of UST use in pediatrics remain an area to improve by conducting large, prospective, randomized, and controlled trials.

**Please acknowledge all funding agencies by checking the applicable boxes below:**

None

**Disclosure of Interest:**

None Declared

